# Supplementary material for: Mode of carbon and energy metabolism shifts lipid composition in the thermoacidophile Acidianus
Source: Appl Environ Microbiol. 2024 Jan 18;90(2):e01369-23. doi: 10.1128/aem.01369-23 (PMC10880624; doi:10.1128/aem.01369-23)
Supplement: Supplemental material — Additional experimental details, supplementary tables and figures. [file aem.01369-23-s0001.docx]

# Supplemental Material

*Acidianus* DS80 was originally isolated from Dragon Spring (78°C, pH 3.1), located in the Hundred Springs Plain area of Norris Geyser Basin in YNP. The initial inoculum for these experiments was obtained from an active pure culture growing anaerobically on H_2_/S^0^ provided by the Boyd lab at Montana State University.

## Preparation of Wolfe’s Vitamins Solution

Wolfe’s vitamins solution was prepared by dissolving the following components in the appropriate volume of ultrahigh purity water: Pyridoxine HCl (0.01g/L), p-aminobenzoic acid (0.005 g/L), lipoic acid (0.005 g/L), nicotinic acid (0.005 g/L), riboflavin (0.005 g/L), thiamine HCl (0.005 g/L), calcium pantothenate (0.005 g/L), biotin (0.002 g/L), folic acid (0.002 g/L), cyanocobalamin (0.0001 g/L). Complete Wolfe’s vitamins solution was filter-sterilized and frozen at -20°C, with one liquid working stock kept in the dark at 4°C.

## Preparation of SL-10 Trace Elements Solution

SL-10 trace elements solution was prepared by dissolving the following components in the appropriate volume of ultrahigh purity water: FeCl_2_ x 4H_2_O (1.5 g/L), ZnCl_2_ (0.07 g/L), MnCl_2_ x 4H_2_O (0.1 g/L), H_3_BO_3_ (0.006 g/L), CoCl_2_ (0.1037 g/L), CuCl_2_ x 2H_2_O (0.002 g/L), NiCl_2_ x 6H_2_O (0.024 g/L), Na_2_MoO_4_ x 2H_2_O (0.036 g/L), Na_2_WO_4_ (0.015 g/L), Na_2_SeO_3_ (0.0098 g/L). The solution was acidified using HCl (33% v/v) at a concentration of 7.5 mL/L. SL-10 trace elements solution was filter-sterilized and stored in the dark at 4 °C.

## Cline assay for tracking S^0^ reduction

The methylene blue method (Fogo and Popowsky, 1949) was used to track sulfide production throughout the course of the experiment. Briefly, 15 μL ferric chloride, 50 μL amine-sulfuric acid, and 750 μL sample (withdrawn from bottles using a 1 mL syringe) were combined in a 1.5 mL centrifuge tube. The solution rapidly turned blue in the presence of dissolved sulfide. Immediately after the color change, 160 μL of diammonium hydrogen phosphate (DHP) was added to quench the reaction, resulting in the formation of a white precipitate. The centrifuge tube was vortexed briefly to dissolve the precipitate. Contents of the centrifuge tube were poured into a 1 mL glass cuvette and absorbance was measured at 670 nm on a Genesys 10S UV-Vis spectrophotometer (Thermo Fisher).

The average dissolved sulfide concentration was calculated from three biological replicates, and the amount of total sulfide produced was calculated assuming standard gas-phase equilibrium. A temperature-corrected Henry’s constant (K_H_) was calculated using a Henry’s constant (K_H_°) of 0.087 and temperature dependence on solubility constant, (–dlnK_H_)/dT, of 2100 (De Bruyn et al., 1995)

## Ferrozine assay for tracking Fe^3+^ reduction

Ferrous iron (Fe^2+^) production was tracked using the ferrozine assay (Viollier et al., 2000). At each timepoint, 300 μL of sample (withdrawn from bottles using a 1 mL syringe) was transferred to a centrifuge tube, and gently spun down at 12,000 × *g* for 30 seconds to separate out inorganic suspended matter. 100 μL of the resulting supernatant liquid was transferred to a centrifuge tube containing 900 μL ferrozine solution. The mixture turned purple in the presence of Fe^2+^. The tube was vortexed briefly to mix, and contents were poured into a 1 mL glass cuvette. Absorbance was measured at 562 nm on a Genesys 10S UV-Vis spectrophotometer (Thermo Fisher).

# Supplementary tables

**Table S1.** Peak areas for GDGT species detected via HPLC-APCI-MS operated in single ion monitoring mode. Ring index is a weighted average of cyclopentane rings in all GDGTs from a sample (see Eqn. 1).

| **Sample** | **GDGT-0** | **GDGT-1** | **GDGT-2** | **GDGT-3** | **GDGT-4** | **GDGT-5** | **GDGT-6** | **Avg.**  **Ring Index** |
| --- | --- | --- | --- | --- | --- | --- | --- | --- |
| S^0^/Fe^3+^ (B1) | 3600  (0.5%) | 1070  (0.1%) | 5881  (0.8%) | 24615  (3.4%) | 262618  (36.5%) | 288135  (40.0%) | 133555  (18.6%) | 4.71 ± 0.02 |
| S^0^/Fe^3+^ (B2) | 861  (0.1%) | 2482  (0.3%) | 3227  (0.4%) | 24833  (3.1%) | 286872  (35.9%) | 328272  (41.1%) | 151953  (19.0%) |  |
| S^0^/Fe^3+^ (B3) | 1720  (0.2%) | 1145  (0.2%) | 8121  (1.1%) | 27221  (3.7%) | 268058  (36.3%) | 299149  (40.5%) | 133727  (18.1%) |  |
| H_2_/Fe^3+^ (B1) | 8126  (1.4%) | 3006  (0.5%) | 20198  (3.6%) | 46080  (8.1%) | 288336  (50.7%) | 162159  (28.5%) | 40561  (7.1%) | 4.10 ± 0.07 |
| H_2_/Fe^3+^ (B2) | 19933  (2.6%) | 13629  (1.8%) | 22052  (2.9%) | 64672  (8.6%) | 406225  (53.8%) | 200677  (26.6%) | 27471  (3.6%) |  |
| H_2_/Fe^3+^ (B3) | 19267  (2.4%) | 13218  (1.6%) | 36534  (4.5%) | 56144  (6.9%) | 437411  (54.0%) | 203314  (25.1%) | 44314  (5.5%) |  |
| H_2_/S^0^/CO_2_ (B1) | 14412  (4.6%) | 14713  (4.7%) | 17423  (5.5%) | 32024  (10.2%) | 111029  (35.3%) | 94416  (30.0%) | 30840  (9.8%) | 3.89 ± 0.12 |
| H_2_/S^0^/CO_2_ (B2) | 11199  (3.7%) | 10420  (3.4%) | 26195  (8.6%) | 29942  (9.9%) | 101001  (33.2%) | 95132  (31.3%) | 29950  (9.9%) |  |
| H_2_/S^0^/CO_2_ (B3) | 12560  (5.5%) | 11297  (4.9%) | 20975  (9.1%) | 33236  (14.4%) | 70639  (30.7%) | 66302  (28.8%) | 15390  (6.7%) |  |
| H_2_/S^0^/glucose (A) | 7655  (2.4%) | 21181  (6.7%) | 63525  (20.0%) | 50793  (16.0%) | 81490  (25.6%) | 65327  (20.6%) | 27754  (8.7%) | 3.61 ± 0.11 |
| H_2_/S^0^/glucose (B) | 6957  (2.4%) | 16888  (5.8%) | 50255  (17.4%) | 43737  (15.1%) | 67372  (23.3%) | 74965  (26.0%) | 28543  (9.9%) |  |

###

**Table S2.** Summary of bioenergetics, metabolic rates, and ring indices across all conditions tested in this study. Rates of metabolites produced and electrons transferred were calculated for data points representing logarithmic growth. Average ring indices were calculated for the biomass harvested during late logarithmic or early stationary phase

| Sample | Time^a^  (hour) | Metabolite production rate^b^  (mmol/L/day) | Avg. metabolite production rate^b^  (mmol/L/day ± s.d.) | Electron  transfer rate^c^  (mmol e^–^/L/day) | Avg. electron  transfer rate^c^  (mmol e^–^/L/day ± s.d.) | Ring Index^d^  (RI) | Avg. Ring Index^d^  (RI ± s.d.) |
| --- | --- | --- | --- | --- | --- | --- | --- |
| S^0^/Fe^3+^/CO_2_ #1 | 136 | 0.38 | 0.39 ± 0.05 | 2.29 | 2.37 ± 0.28 | 4.70 | 4.71 ± 0.03 |
| S^0^/Fe^3+^/CO_2_ #1 | 185 | 0.42 |  | 2.51 |  |  |  |
| S^0^/Fe^3+^/CO_2_ #2 | 136 | 0.36 |  | 2.15 |  | 4.74 |  |
| S^0^/Fe^3+^/CO_2_ #2 | 185 | 0.46 |  | 2.79 |  |  |  |
| S^0^/Fe^3+^/CO_2_ #3 | 136 | 0.33 |  | 1.99 |  | 4.69 |  |
| S^0^/Fe^3+^/CO_2_ #3 | 185 | 0.41 |  | 2.48 |  |  |  |
| H_2_/Fe^3+^/CO_2_ #1 | 136 | 0.27 | 0.38 ± 0.08 | 0.53 | 0.77 ± 0.16 | 4.20 | 4.10 ± 0.09 |
| H_2_/Fe^3+^/CO_2_ #1 | 185 | 0.36 |  | 0.72 |  |  |  |
| H_2_/Fe^3+^/CO_2_ #2 | 136 | 0.35 |  | 0.70 |  | 4.03 |  |
| H_2_/Fe^3+^/CO_2_ #2 | 185 | 0.46 |  | 0.93 |  |  |  |
| H_2_/Fe^3+^/CO_2_ #3 | 136 | 0.39 |  | 0.77 |  | 4.06 |  |
| H_2_/Fe^3+^/CO_2_ #3 | 185 | 0.48 |  | 0.96 |  |  |  |
| H_2_/S^0^/CO_2_ #1 | 136 | 0.43 | 0.44 ± 0.16 | 0.87 | 0.87 ± 0.32 | 3.96 | 3.89 ± 0.14 |
| H_2_/S^0^/CO_2_ #1 | 185 | 0.70 |  | 1.40 |  |  |  |
| H_2_/S^0^/CO_2_ #2 | 136 | 0.30 |  | 0.60 |  | 3.99 |  |
| H_2_/S^0^/CO_2_ #2 | 185 | 0.53 |  | 1.06 |  |  |  |
| H_2_/S^0^/CO_2_ #3 | 136 | 0.26 |  | 0.51 |  | 3.73 |  |
| H_2_/S^0^/CO_2_ #3 | 185 | 0.40 |  | 0.81 |  |  |  |
| H_2_/S^0^/Glucose A | 96 | 0.05 | 0.10 ± 0.04 | 0.11 | 0.20 ± 0.09 | 3.52 | 3.61 ± 0.11 |
| H_2_/S^0^/Glucose A | 121 | 0.12 |  | 0.24 |  |  |  |
| H_2_/S^0^/Glucose B | 96 | 0.08 |  | 0.17 |  | 3.69 |  |
| H_2_/S^0^/Glucose B | 121 | 0.15 |  | 0.29 |  |  |  |

^a^ For each experiment, two consecutive time points representative of logarithmic growth were selected. The average of rates calculated from biological replicates at these two time points were reported as the average metabolite production rate and average electron transfer rate in Table 2.

^b^ Rates of Fe^2+^ production per liter for the S^0^/Fe^3+^/CO_2_ and H_2_/Fe^3+^/CO_2_ conditions, based on the Ferrozine assay results; rate of S^2–^ production per liter for the H_2_/S^0^/CO_2_ condition, based on the Cline assay results.

^c^ Rates of electrons transferred per liter of culture, based on metabolite production rates and reaction stoichiometries in Eqn. 2–4.

^d^ Ring indices were calculated from biomass harvested during late logarithmic or early stationary phase.

# Supplementary figures

**Figure S1.** Metabolite production during the growth of DS80. Changes in the concentration of [Fe^2+^] during growth on S^0^/Fe^3+^/CO_2_ (A) and H_2_/Fe^3+^/CO_2_ (B). Changes in the concentration of [S^2–^] during growth on H_2_/S^0^/CO_2_ (C) and H_2_/S^0^/glucose (D). For Fe^3+^-reducing conditions (A and B), the absorbance measured via the ferrozine assay was used to calculate [Fe^2+^]. For S^0^-reducing conditions (C and D), the absorbance measured via the methylene blue assay and standard gas-phase equilibrium calculation were used to estimate total sulfide concentration or [S^2–^]. Biomass was harvested immediately after the final time point, when it was determined that cultures reached an early stationary phase.

**Figure S2**. Average ring index values as a function of cell-specific electron transfer rate. The rates were calculated from the measured rates of Fe^2+^ or S^2–^ production per liter of culture, reaction stoichiometries in Eq. 2–4, and the growth yield inferred from the data reported in Amenabar et al. (2017). The same growth yield value for the H_2_/S^0^/CO_2_ treatment in Amenabar et al. (2017) was used for both the H_2_/S^0^/CO_2_ and H_2_/S^0^/glucose treatments in this study.
